# Bioelectric Signaling Regulates Size in Zebrafish Fins

**DOI:** 10.1371/journal.pgen.1004080

**Published:** 2014-01-16

**Authors:** Simon Perathoner, Jacob M. Daane, Ulrike Henrion, Guiscard Seebohm, Charles W. Higdon, Stephen L. Johnson, Christiane Nüsslein-Volhard, Matthew P. Harris

**Affiliations:** 1 Max Planck Institute for Developmental Biology, Tübingen, Germany; 2 Orthopedic Research Laboratories, Boston Children's Hospital; Department of Genetics, Harvard Medical School, Enders, Massachusetts, United States of America; 3 Abteilung Myozelluläre Elektrophysiologie, Institut für Genetik von Herzerkrankungen, Universitätsklinikum Münster, Albert-Schweizer-Campus 1, Münster, Germany; 4 Department of Genetics, Washington University Medical School, St. Louis, Missouri, United States of America; University of Pennsylvania School of Medicine, United States of America

## Abstract

The scaling relationship between the size of an appendage or organ and that of the body as a whole is tightly regulated during animal development. If a structure grows at a different rate than the rest of the body, this process is termed allometric growth. The zebrafish *another longfin (alf)* mutant shows allometric growth resulting in proportionally enlarged fins and barbels. We took advantage of this mutant to study the regulation of size in vertebrates. Here, we show that *alf* mutants carry gain-of-function mutations in *kcnk5b*, a gene encoding a two-pore domain potassium (K^+^) channel. Electrophysiological analysis in *Xenopus* oocytes reveals that these mutations cause an increase in K^+^ conductance of the channel and lead to hyperpolarization of the cell. Further, somatic transgenesis experiments indicate that *kcnk5b* acts locally within the mesenchyme of fins and barbels to specify appendage size. Finally, we show that the channel requires the ability to conduct K^+^ ions to increase the size of these structures. Our results provide evidence for a role of bioelectric signaling through K^+^ channels in the regulation of allometric scaling and coordination of growth in the zebrafish.

## Author Summary

The proportions of an animal can change during its lifetime. This often occurs through the phenomenon of relative growth, or allometry. In humans, for example, the head grows at a lower rate than the body resulting in a change in proportion between children and adults. The regulation of size and proportion is not well understood. We investigated fin growth in zebrafish as a model to understand this phenomenon. The mutant *another longfin* develops proportionally larger fins and barbels. Here, we show that *another longfin* mutants carry a mutation in *kcnk5b*, a gene coding for a potassium channel. Potassium channels control the electrical potential of cells and are known to regulate processes such as heart beat, neurotransmission and hormone secretion. We demonstrate that increased potassium channel activity can drive changes in growth in the zebrafish. Clonal analysis reveals that the channel acts directly in the fins and barbels to increase their size in a coordinated manner. Our work shows that potassium channels are involved in the determination of appendage size in zebrafish and suggests consistent with recent studies, an instructive role for bioelectrical signaling in development.

## Introduction

Organ growth is a complex process that requires attaining not only a certain shape but also an appropriate size. The maintenance of proper proportions between organs is tightly regulated [1]. The growth of a structure at a different rate with respect to the rest of the body results in changes in proportions during development. Such allometric growth accounts for the morphological differences between juvenile and adult stages in numerous organisms. This process also contributes to changes in shape and morphology during evolution [2], [3].

Growth is regulated by both organ-intrinsic signals as well as growth factors and hormones that originate outside the target organ. Their relative contribution can vary depending on the species or even between different structures within the same organism [4], [5]. Analysis of chimeras, obtained from transplantation experiments during embryonic stages, has shown that in many cases the final size of an organ is independent of extrinsic factors, such as nutrients or hormones, suggesting that determination of size and shape are organ-autonomous properties [6]. For instance, reciprocal xenografts of limb buds between salamander species of different sizes lead to limbs that attain the final size of the donor species [7]. Further, grafting experiments in avian models have shown that the mesenchyme harbors the instructive information that specifies the final size and shape of structures such as the limb and the beak [8]–[11].

The final size of an organ or appendage results from a combination of cell number and cell size. Perturbation of the Hippo pathway causes massive proliferation of *Drosophila* tissues and tumorigenesis in mouse [12], while hyperactivation of the TOR pathway stimulates cell growth and can trigger entry into the cell cycle [13]. Locally acting molecules such as insulin-like growth factors (IGFs) and fibroblast growth factors (FGFs) are essential regulators of growth [6]. Yet, how these components are integrated to establish proper patterning and size during development as well as during regeneration is still unclear.

Two-pore domain potassium (K_2P_) channels are a family of potassium (K^+^) channels that play an important role in determining membrane potential and cell excitability [14]. These leak K^+^ channels conduct instantaneous currents that are independent of voltage and show open rectification, i.e. they mediate primarily outward currents under physiological conditions. K_2P_ channel function is modulated by neurotransmitters and pharmacological compounds as well as physiological parameters such as temperature, oxygen, osmolarity and pH [15]. Due to their ability to respond to multiple biological stimuli and their wide expression across tissues, they are thought to control many physiological processes besides determining the membrane potential. Although these ion channels have not been implicated in organ size control so far, evidence has been accumulating that endogenous bioelectrical signals orchestrate patterning and growth [16]. Endogenous electrical currents are associated with limb development and regeneration in vertebrates [17], [18] and changes in voltage accompany cessation of regenerative growth in earthworms [19]. In *Xenopus laevis*, a species with limited regenerative capacity, artificial induction of currents can enhance the regeneration process [20], [21], while chemical, pharmacological or molecular inhibition of ionic currents can abrogate regeneration in this species [22]–[24].

Fins are structures that show an enormous diversity in shape and size in different fish species. They also possess a remarkable regenerative capacity [25]; they can easily be manipulated and unlike internal organs, fins do not have obvious limitations on growth. The skeleton of zebrafish fins consists of a proximal endochondral and a distal dermal skeletal component. The latter is formed by segmented, concave fin rays, the lepidotrichia, which originate from mesenchymal condensations [26]. Fins grow through sequential addition of lepidotrichial segments at their distal tip via migration of mesenchymal cells along the actinotrichia, clusters of collagenous fibers that emerge from the tip of each lepidotrichium [27], [28]. Segment length slightly decreases along the proximo-distal axis [26], but does not change once joints are formed and segment boundaries are established [29]. In zebrafish numerous fin mutants have been isolated over the years [30]–[33]. Most of these mutants have reduced fins [34]. For example, impairment of the ectodysplasin signaling causes loss of fin rays in *finless* and *nackt* mutants [35], while in *short fin (sof)* mutants defects in connexin 43 *(cx43)* lead to decreased fin size with shorter segments [36]. A few mutants exhibit increased allometric growth of the fin. Among these, *longfin (lof)* and *rapunzel (rpz)* mutants have an increased number of ray segments [32], [37], whereas *another longfin (alf)* mutants tend to have elongated segments [36]. So far, the genetic lesion has only been identified for *rpz*, which is mutated in a novel teleost-specific gene with unknown function [38].

Here, we report that the allometric fin overgrowth displayed by *alf* mutants is due to the altered function of Kcnk5b, a K_2P_ channel. Our analysis indicates that mutant Kcnk5b acts locally within the mesenchyme of fins and barbels to increase appendage size. Furthermore, we demonstrate that K^+^ conductance is required to cause allometric growth during development. Genetic experiments suggest that *kcnk5b* may act independently of, or in parallel to, *cx43*. Taken together our results provide *in vivo* evidence for a role of K^+^ channels in the determination of appendage size and proportion in the zebrafish.

## Results

### *alf* mutants display increased growth and proportion of appendages

**Figure 1:**
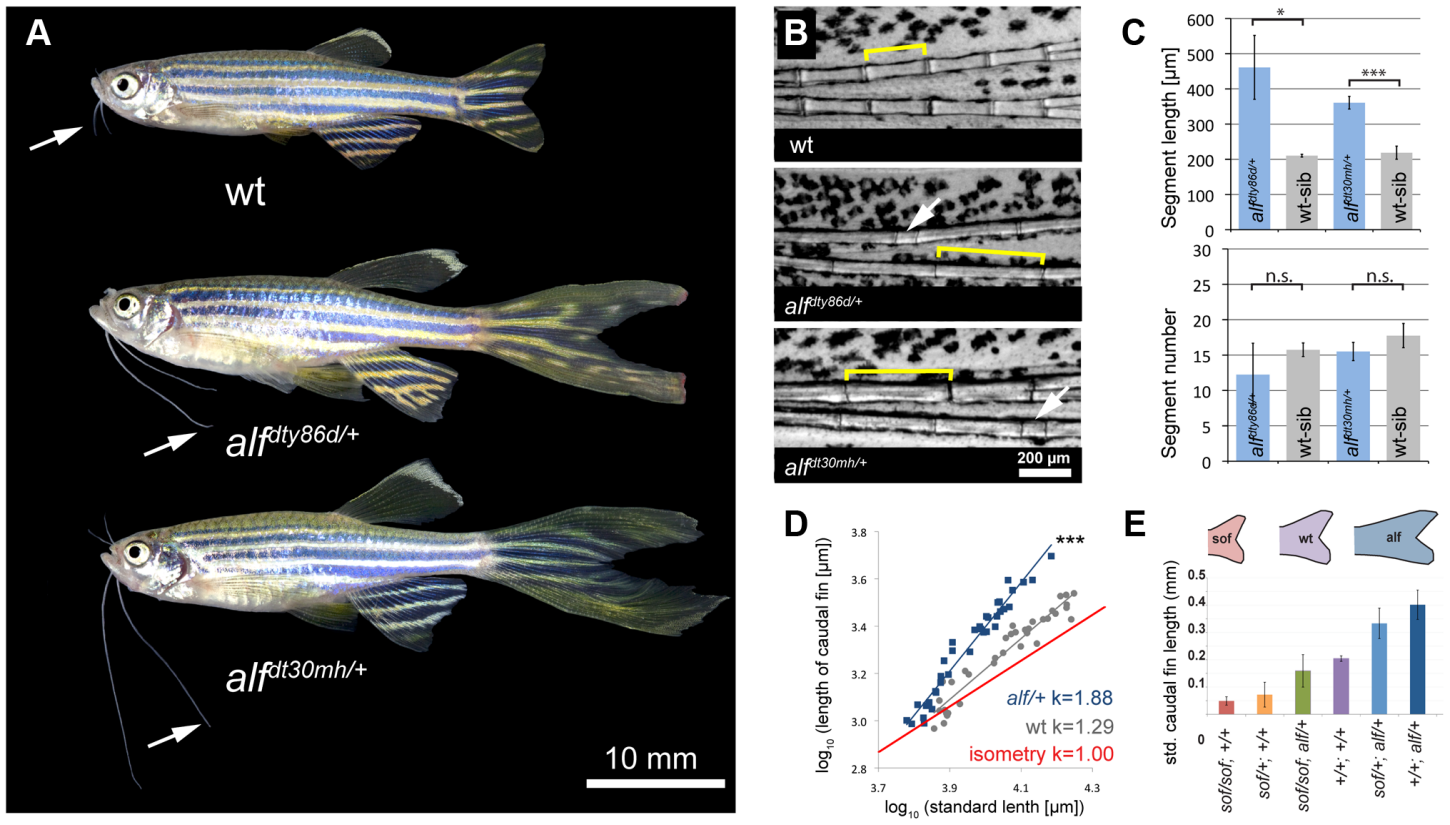
*alf* mutants lead to an increase in size of the appendages of adult fish. (A) *alf* mutations are dominant and lead to overgrown fins and barbels in the adult. Arrows indicate maxillary barbels; the mutants shown are heterozygous. (B) Segment patterning in the dorsal fin of wild type and heterozygous mutants. Brackets indicate one segment. Although the majority of segments show increased length, several short segments can be seen in the mutants (arrows). (C) Variation in segment length (top) and segment number (bottom) in the longest ray of the dorsal fin of mutants and wild type siblings (wt sib). Fish of similar standard length (SL) (i.e. distance between snout and caudal peduncle) were compared; all cases n = 4; error bars: standard deviation; n.s.: not significant; *: p<0.02, ***: p<0.001. (D) Increased allometric scaling of heterozygous *alf* fins in development. *k* = allometric coefficient, Linear regression lines, wt R^2^ = 0.92; *alf/+*, R^2^ = 0.95; ***: p<0.001. (E) Crosses of *sof* with *alf* indicate that there is not epistatic interaction between the two genes. Fin length was normalized with SL.

*another longfin* (*alf^dty86d^*) was identified in a large-scale mutagenesis screen as a mutant with elongated fins and irregular segmentation of the fin rays [30], [34]. In a subsequent mutagenesis screen we isolated a second mutation (*alf^d30mh^*) showing an identical phenotype and mapping to the same chromosomal region as the original *alf* allele (see below). Besides the longer fins, *alf* mutants show overgrowth of the barbels, (Figure 1A, arrows). Homozygous mutants have a stronger phenotype (Figure S1) and their fins tend to be particularly susceptible to breakage leading to accretion of bone around the lesions. Overgrown fins and barbels in *alf* mutants retain their general organization; however, the fins have an altered segmentation pattern, as joint formation is variable in the mutants. On average, the length of lepidotrichial segments is increased [36] (Figure 1B and 1C); however, structures appearing as very short segments are occasionally observed (arrows in Figure 1B). In contrast to other fin overgrowth mutants such as *lof* or *rpz* [32], [37], the number of segments is not increased in *alf* mutants (Figure 1C).

**Figure 2:**
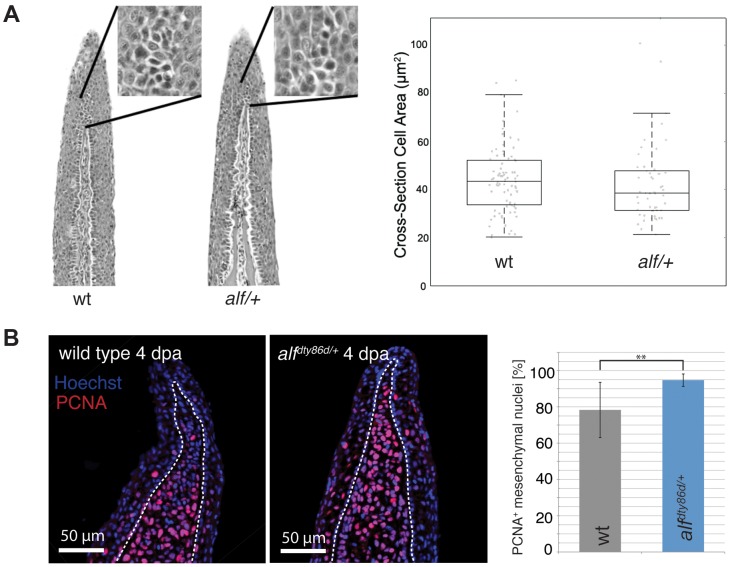
Cell proliferation is increased in *alf* mutants. (A) Sections of wild type and heterozygous *alf* fins. No significant difference in cell size is seen in the two groups. (B) Antibody staining against PCNA on paraffin sections of regenerating fins 4 days post amputation (dpa). Chart shows percentage of proliferating nuclei (PCNA) over total nuclei (Hoechst). N = 3–4 sections of 4 individual fish **: p-value<0.01.

Analysis of the caudal fins during development showed that the increase in size seen in the mutants is due to an increased growth rate (Figure 1D). Wild type (wt) fins exhibit only a slight increase in relative growth during development (*k* = 1.29) as growth is essentially isometric [32]. *alf* heterozygotes showed positive allometric growth during development of the fin with an allometric coefficient *k* near 2 (Figure 1D). Histological analysis of fins from heterozygous fish does not reveal appreciable differences in the size of scleroblasts and epidermal cells over those seen in wild type sections (Figure 2A). However, increased staining of the proliferating cell nuclear antigen (PCNA) during fin regeneration suggests that proliferation is increased in the mutants (Figure 2B).

In *sof* mutants defects in *cx43* are known to cause a reduction in both fin size and segment length [36]. We therefore tested whether the *alf* overgrowth phenotype requires the function of *cx43*. Crosses between *alf^dt30mh^* and a dominant *sof* allele, *sof^dj7e2^*, showed no epistatic interaction between the two genes (Figure 1E), suggesting that the two mutations most likely affect independent processes that both contribute to the determination of final appendage size during fin development.

### The *alf* phenotype is due to missense mutations in *kcnk5b*

**Figure 3:**
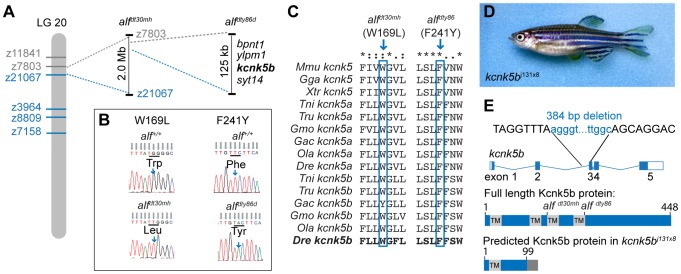
The *alf* phenotype is due to gain-of-function mutations within the K^+^ channel *kcnk5b*. (A) *alf* mutations map to chromosome 20 between z11841 and z21067. *Gray*: north markers; *blue*: south markers. (B) Electropherogram of *kcnk5b* at position 169 and 241 in mutants and wild type siblings. (C) The amino acids affected in the mutants are well conserved among vertebrates. (D) A revertant of *alf^dty86d^* (*j131x8*) shows wild type-sized fins. (E) *kcnk5b^j131x8^* fish harbor an intragenic deletion in *kcnk5b* that is predicted to cause a truncated protein lacking three transmembrane (TM) domains.

We mapped the *alf* mutations to overlapping regions on chromosome 20 (Figure 3A). We further refined *alf^dty86d^* to a genomic interval of 125 kb coding for 4 genes (*bpnt1*, *ylpm1*, *kcnk5b*, and *syt14*). In both *alf* alleles, distinct missense mutations (W169L and F241Y) were identified in the coding sequence for *kcnk5b* (Figure 3B). This gene encodes a K_2P_ channel. The affected residues are highly conserved in *kcnk5b* homologs of other vertebrate species (Figure 3C). Thus, the *alf* phenotype is due to allelic mutations in *kcnk5b*.

**Figure 4:**
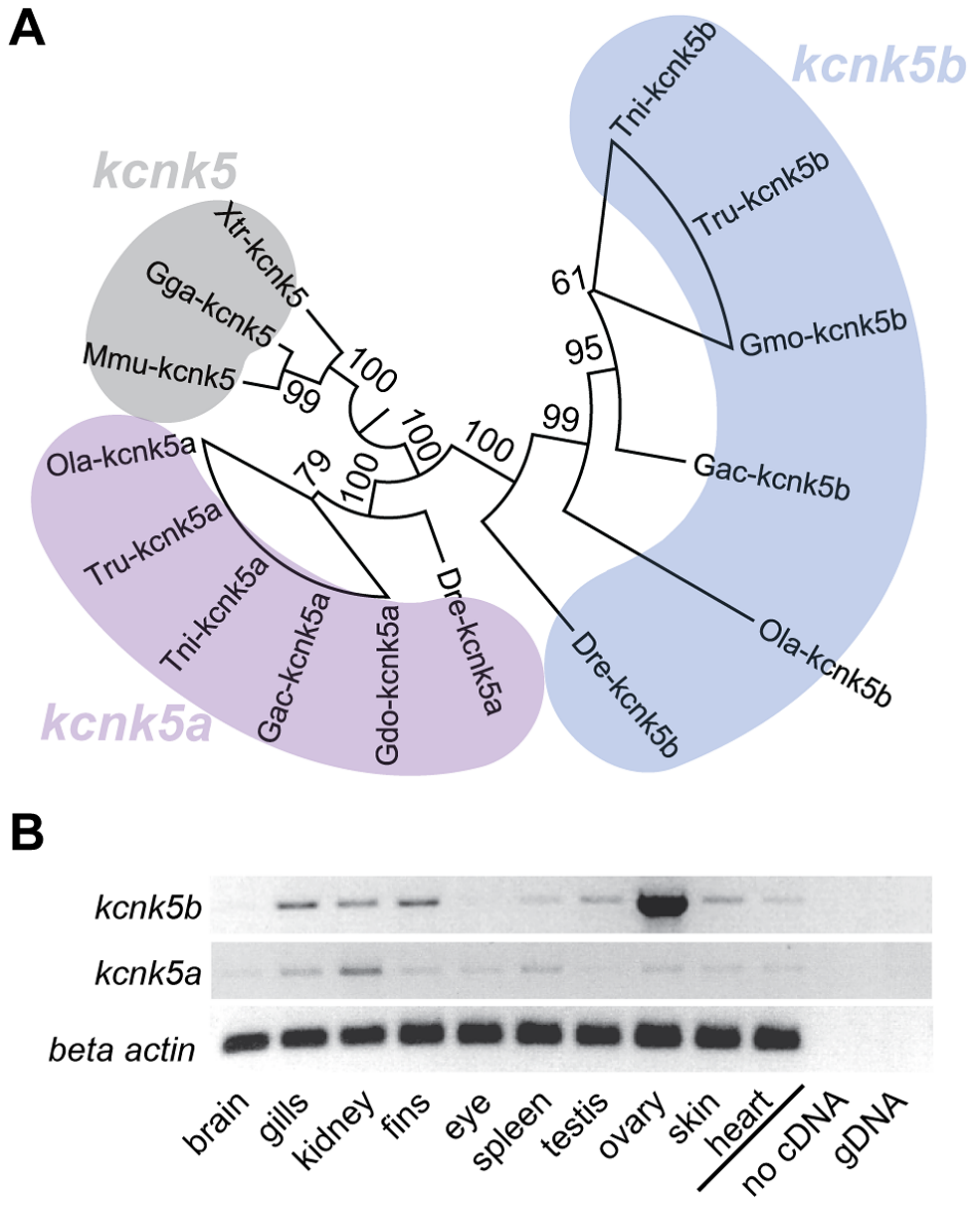
Vertebrate *kcnk5* homologs and expression in zebrafish development. (A) Due to a whole genome duplication event, teleost fish have two *kcnk5* paralogs that show early divergence. Numbers indicate bootstrap values in percentage (100 bootstrap replications). Nodes with a bootstrap value lower than 95 were collapsed. Dre, *Danio rerio*; Ola, *Oryzias latipes*; Gac, *Gasterosteus aculeatus*, Tru, *Takifugu rubripes*; Tni *Tetraodon nigridoviridis*, Gmo, *Gadus morhua*; Mmu, *mus musculus*; Gga, *Gallus gallus*; Xtr *Xenopus tropicalis*. (B) RT-PCR of *kcnk5a* and *kcnk5b* shows comparable expression between the two paralogs in multiple adult tissues, including fins.

To assess the nature of these alleles we generated a phenotypic revertant *(j131x8)* of the dominant *alf^dty86d^* mutant (Figure 3D). PCR analysis of genomic DNA showed the presence of a 384 bp deletion leading to a frameshift and a premature termination codon. The resulting protein is predicted to lack 3 of the 4 transmembrane (TM) domains (Figure 3E). This suggests that the revertant is a null mutation for *kcnk5b*. Homozygotes harboring the deletion are viable and fertile; thus, *kcnk5b* is not essential for zebrafish development. As *kcnk5b* has a close paralog in zebrafish, *kcnk5a* (Figure 4A), which is expressed in similar tissues (Figure 4B), the lack of a loss-of-function phenotype in normal development may be due to functional redundancy between the paralogs. Together, these data endorse our finding that *kcnk5b* is the gene responsible for the *alf* overgrowth phenotype and demonstrate that these mutations are due to a gain of function rather than haploinsufficiency.

### *alf* mutations in *kcnk5b* lead to increased K^+^ conductance and hyperpolarization

**Figure 5:**
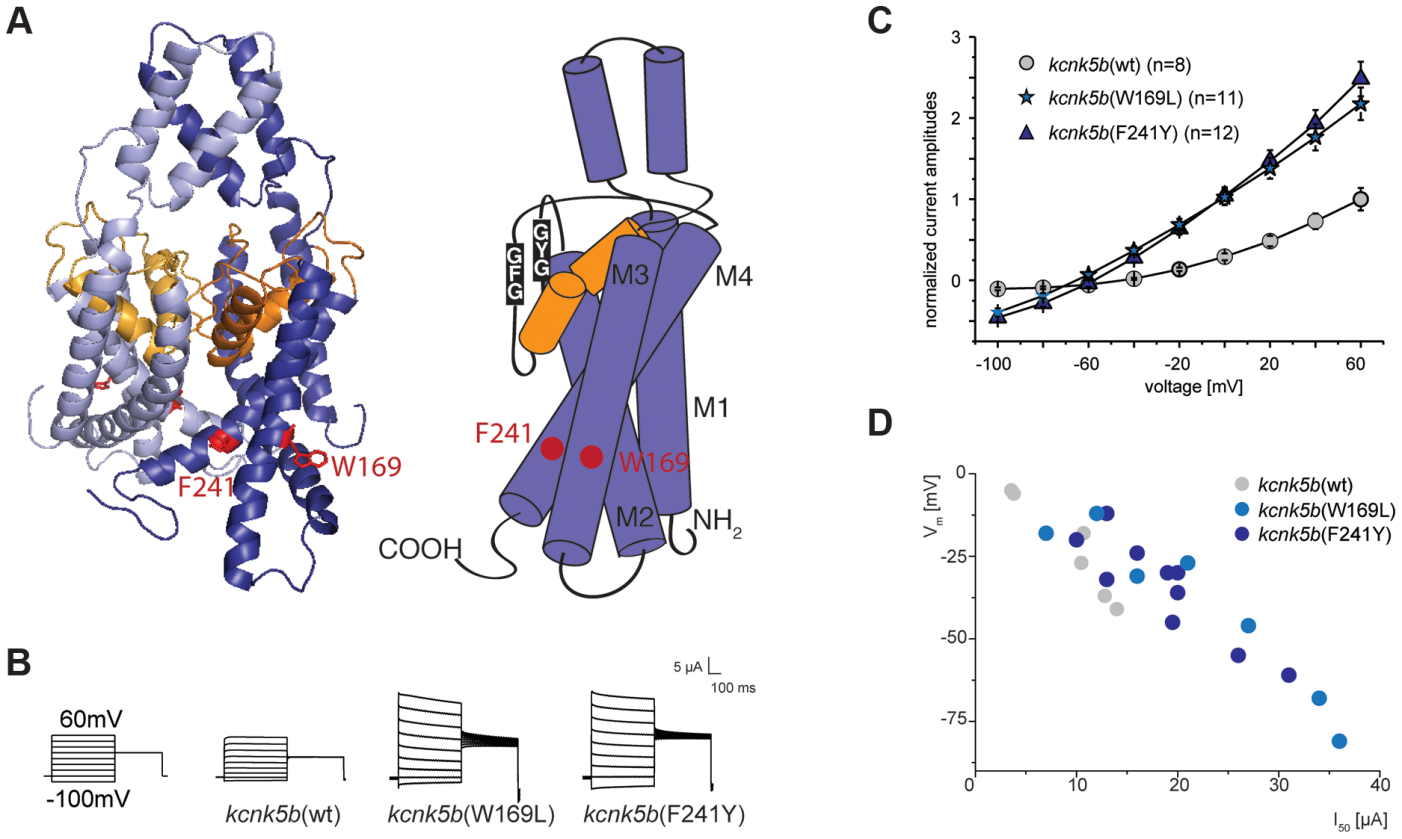
Gain-of-function mutations in *kcnk5b* affect ionic conduction and lead to hyperpolarization of the cell. (A) Location of the amino acids altered in *kcnk5b* gain-of-function mutants. Kcnk5b protein was modeled on human KCNK4 (K2p4.1). GFG and GYG domains represent the selectivity pore of the channel. (B) Voltage clamp recordings from *Xenopus* oocytes injected with cRNA of wild type and mutant *kcnk5b*. The membrane potential was clamped at a reference potential of −80 mV and then stepped to a test potential from +60 mV to −100 mV for 500 ms. The current that is applied in order to clamp the voltage to a certain value corresponds to the current passing through the plasma membrane. Representative electrophysiological traces are shown. (C) The mutant channels display increased conductance over wild type channels expressed at comparable levels. Error bars represent standard deviation. (D) Kcnk5b influences membrane potential (V_m_) in oocytes. The mutant variants tend to hyperpolarize the cell (each point represents one oocyte).

We used the known structure of human KCNK4 (K2p4.1) [39] as a template for modeling Kcnk5b and assessing the mutations. These models revealed that the affected amino acids are positioned in two distinct TM domains towards the cytoplasmic side of the protein (Figure 5A).

To assess how the identified amino acid substitutions might affect Kcnk5b function, the channel properties were tested in a two-electrode voltage clamp experiment in *Xenopus* oocytes. This technique permits measurement of currents across the cell membrane when the membrane potential is clamped to a given value. Oocytes injected with *kcnk5b*(wt) cRNA react steadily to a change in voltage and do not exhibit a delay in current flow, as is expected for a K_2P_ channel. A similar situation is also seen with *kcnk5b*(W169L) or *kcnk5b*(F241Y) cRNAs. However, oocytes injected with either of both mutant cRNAs show an almost two-fold increase in K^+^ conductance over that of oocytes injected with wild type cRNA (Figure 5B). The current-voltage relationship of the wild type channel shows the typical outward rectification of a K_2P_ channel, i.e. current flows preferentially out of the cell, from the side of high K^+^ concentration to the side of low K^+^ concentration [40]. In contrast, the increase in K^+^ currents in the Kcnk5b mutant variants is accompanied by reduced outward rectification (Figure 5C) suggesting that the change in K^+^ conductance results from altered biophysical features of Kcnk5b rather than a simple increase in the number of channels at the plasma membrane.

K_2P_ channels are often referred to as leak channels since they account for the constant leaking current that sets the resting membrane potential observed in neurons. They are known to control both cell excitability and membrane potential [41], and the human homolog of *kcnk5b*, *KCNK5* (TASK2), was shown to contribute significantly to the stabilization of the membrane potential in articular chondrocytes [42]. Therefore, we hypothesized that zebrafish Kcnk5b might also play a role in setting the membrane potential. Indeed, the membrane potential values of oocytes injected with wild type and mutant *kcnk5b* cRNAs are correlated with the amplitude of the ion current measured at a constant voltage of 50 mV (Figure 5D): the higher the conductance for K^+^ measured at 50 mV, the more negative the membrane potential of the oocyte. Consistently, the mutant channels lead to stronger hyperpolarization causing a shift in the membrane potential towards −90 to −100 mV, the equilibrium potential for K^+^ in *Xenopus* oocytes.

### *kcnk5b* acts locally to increase appendage size

**Figure 6:**
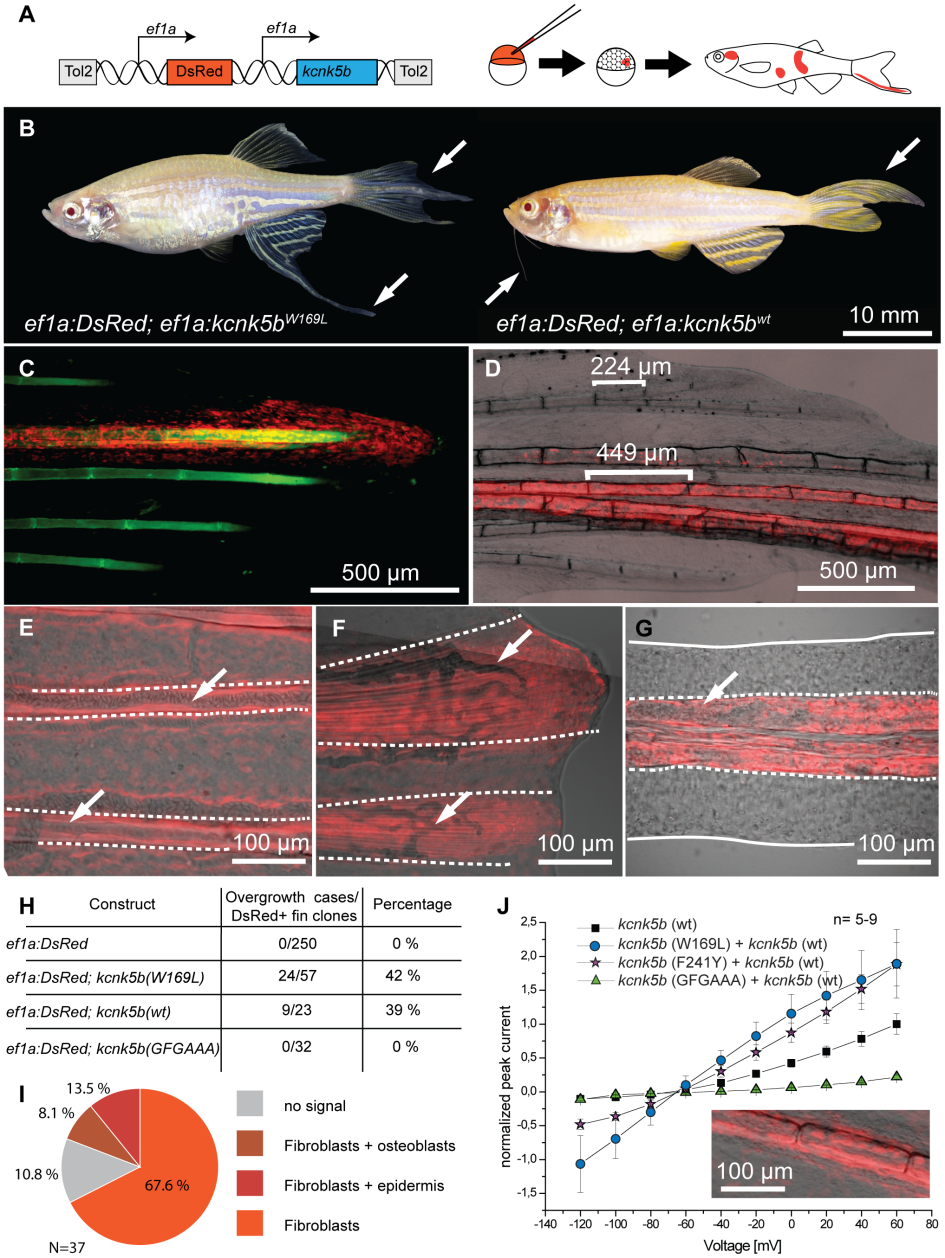
Overexpression of *kcnk5b* is sufficient to cause fin overgrowth. (A) Construct used to create *kcnk5b*-expressing clones via Tol2 transgenesis. (B) Individual fish expressing *kcnk5b* (W169L) (left) or *kcnk5b* (wt) (right) in mosaic clones display localized fin and barbel overgrowth. (C–F) Overgrowth is associated with DsRed expression (in red) within mesenchymal cells. (C) Calcein staining labels bone tissue (in green) of an overgrown fin (DsRed; *kcnk5*(W169L) expressing clone). (D) Mesenchymal clones are associated with increased segment length in the fin compared to non-overgrown DsRed negative regions. (E) Fibroblast-like cells appear as DsRed positive cells within the fin rays (dotted line) that surround DsRed negative vasculature (arrows in E and F) which extend along the actinotrichia (fibrils within dotted lines in F) towards the distal end of the fin. (G) Overgrown barbels show DsRed signal within the mesenchyme (area within dotted line) but not in the vasculature (arrow). (H) Number of clones associated with overgrowth in different *kcnk5b* variants. (I) Proportion of different cell types labeled in overgrown tissues. (J) Electrophysiological recordings of the non-conductive *kcnk5b* (GFGAAA) mutant in oocytes. Squares: *kcnk5b* (wt), purple stars: *kcnk5b* (F241Y)+*kcnk5b* (wt), blue circles: *kcnk5b* (W169L)+*kcnk5b* (wt), green triangles: + *kcnk5b* (GFGAAA)+*kcnk5b* (wt). Current was normalized to the measurement of wt current at 60 mV. Inset: DsRed+ fibroblasts in fish injected with the non-conductive construct do not lead to fin overgrowth.

To show where *kcnk5b* is expressed we performed *in situ* hybridization experiments on adult fins, however no specific signal above background was observed, indicating that expression levels might be below detection with this technique. Nevertheless, RT-PCR analysis showed that *kcnk5b* is expressed in fins of adult fish (Figure 4B). To assess whether *kcnk5b* acts locally within fins and barbels to control growth, we transplanted *kcnk5b^dt30mh/+^* mutant cells into wild type hosts (Figure S2A). Local overgrowth of these structures was detected in 29 out of 120 chimeras raised to adulthood (Figure S2B–D), whereas global overgrowth of all fins and barbels was never observed. This suggests that the mutations act locally within the appendages to increase their size. We further attempted to induce the mutant phenotype by local overexpression of the channel within fins and barbels of wild type fish. Whereas the electrophysiological analysis indicated that the dominant *kcnk5b* mutations lead to an increase of channel conductance, the current of K^+^ ions through the plasma membrane depends not only on individual channel conductance, but also on the number of channels present in the membrane. Therefore, we argued that increasing the number of channels should also promote fin overgrowth. We generated a construct in which either *kcnk5b*(wt) or *kcnk5b*(W169L) expression is driven by the elongation factor 1 alpha (*ef1a*) promoter from *Xenopus laevis*; this promoter was recently shown to be active in all major fin tissues [43]. To mark the cells that express the transgene, DsRed expression was driven under a second *ef1a* promoter positioned in *cis* within the same plasmid (Figure 6A). This plasmid was injected into wild type one-cell stage zebrafish zygotes along with Tol2 transposase mRNA as described before [43]. Injected fish were raised to adulthood, screened for DsRed positive cells in the fins and the effects on growth were recorded. No overgrowth was observed in fish injected with a control plasmid expressing only DsRed under the *ef1a* promoter (0/240), despite the presence of DsRed-positive cells in various tissues within the fin (Figure S3). In about 40% of the fish injected with plasmids encoding wild type or mutant *kcnk5b* and showing DsRed positive cells in the fins we found a local overgrowth phenotype (Figure 6B and H). Analysis showed a strong correlation of overgrowth with DsRed positive mesenchymal tissue (89.2%, N = 37, Figure 6C, D and I), whereas DsRed positive cells in other tissues were not associated with increases in size. The fin ray segments were enlarged in the overgrown fins similar to *alf* mutants (Figure 6D). The marked fibroblast-like cells typically occupied the intra-ray space and were excluded from the arteries (Figure 6E). These vessel-surrounding clones extended along the actinotrichia to the distal ends of the overgrown fins (Figure 6F). In the case of barbel overgrowth, DsRed positive cells were found in the mesenchymal tissue surrounding the central rod (Figure 6G), an acellular, non-cartilaginous, non-mineralized structure that supports this organ [44]. In a few cases no DsRed fluorescence signal could be detected within or next to overgrown fin tissue (*kcnk5b*(W169L), 2/26; *kcnk5b*(wt), 2/11), probably due to variegation of promoter activity [43]. In conclusion, these findings indicate that *kcnk5b* overexpression within fibroblasts of the mesenchyme is sufficient to induce fin outgrowth.

To test whether *kcnk5b*-induced overgrowth requires conductance of K^+^ ions by the channel, we generated an overexpression construct encoding a non-conductive version by mutating the GFG motif of the selectivity filter to AAA, *kcnk5b*(GFGAAA). This modification has previously been shown to block ion conductance in K^+^ channels [45]. Electrophysiological measurements in *Xenopus* oocytes showed that this channel is unable to conduct K^+^ (Figure 6J). The plasmid was injected into wild type embryos along with Tol2 transposase mRNA and injected fish were reared to adulthood and assessed for overgrowth. No overgrowth was detected in these fish (Figure 6H), although fins containing DsRed positive tissue (n = 32), including fibroblasts (Figure 6J, inset), were found. These data indicate that the increase in conductance of the Kcnk5b channel is essential for the coordinated overgrowth of the fins and barbels in the mutants.

## Discussion

K^+^ channels have long been associated with neurological function, hormone secretion, and cardiomyocyte polarization [46]. They are a diverse class of ion channels, which can be grouped into four major families: inwardly rectifying (K_IR_), voltage-dependent (K_V_), calcium-dependent (K_Ca_) and two-pore domain (K_2P_) potassium channels. K_IR_ channels have recently been shown to be involved in patterning in vertebrates and invertebrates. In *Drosophila* loss-of-function mutations in *Irk2* lead to wing patterning defects [47]. Mutations in the human homolog, Kir2.1, are associated with craniofacial and digital defects [48]. In zebrafish establishment of the adult pigmentation pattern requires the function of Kcnj13 (Kir7.1) [49]. Here, we report that gain-of-function mutations in *kcnk5b*, a gene encoding a K_2P_ channel, lead to allometric overgrowth of the fins. This is the first time that a member of this class of channels is shown to be involved in regulation of growth and patterning in a vertebrate.

### Implications of K^+^ channels in growth and proliferation

The size of an organ depends on cell size and cell number. The mammalian homolog of *kcnk5b* has been implicated in both, regulation of cell volume [50], [51] and cell proliferation [52], [53]. In *alf* mutants we could detect an increase in cell proliferation but not in cell size (Figure 2). Importantly, the mutant phenotype does not arise simply by dysregulation of cell proliferation, which would cause tumorous overgrowth; rather the overgrown structures in the mutants preserve tissue organization and patterning.

It is unclear how K^+^ channels regulate proliferation. Studies have proposed that this might occur through regulation of the membrane potential [54]. In apparent contrast to some studies [55]–[58] but in agreement with others [59], [60], we found that hyperpolarization caused by mutations in a K^+^ channel can lead to tissue overgrowth. Although we observed a hyperpolarizing effect of the *alf* mutation in *Xenopus* oocytes, we cannot exclude that this, in turn, triggers a depolarization, either at cellular level or in the surrounding tissues during development of the fin. In fact, experiments employing depolarization-sensitive dyes, suggest that this might indeed be the case (Figure S4). The importance of hyperpolarization during growth is supported by regeneration studies in *Xenopus* [22], [24]. Regenerating tadpole tails are initially depolarized, but, unlike tails in the refractory state, subsequently undergo hyperpolarization. Notably, impairing hyperpolarization through inhibition of V-ATPase activity leads to a reduction of cell proliferation and failure to regenerate [22]. Transient hyperpolarization of the cell might lead to a cytosolic increase of the second messenger Ca^2+^, activate integrin-dependent or PTEN phosphatase-dependent cascades, or favor the uptake of mitogens such as serotonin through voltage-dependent transporters [61]. Recent reports suggest that in some cases K^+^ channels can induce cell proliferation independently of their effect on membrane potential [62], [63]. We show that, in the case of Kcnk5b, conductance is essential for the regulation of fin growth. Overexpression of a non-conducting version of the channel does not cause a phenotype, whereas wild type and *alf* variants induce local overgrowth.

### Role of *kcnk5b* in size specification

Our analysis of transgenic mosaics indicates that cells of the mesenchyme are sufficient to provide cues that alter the size of the fins. This is consistent with results of classic xenograft studies between chicken and quail where cells of the mesenchyme impart donor-specific characteristics to the limbs [8], [64]. During development tetrapod limbs are patterned by signaling interactions between mesenchymal cells and the overlying ectoderm. A prominent signaling center, the apical ectodermal ridge (AER), is active at the distal tip of the limb bud during this process. The AER and the mesenchyme of the progress zone continuously communicate with each other to direct limb outgrowth and development. Similar epithelial-mesenchymal interactions from the apical fin fold are likely to be required for the patterned overgrowth of fins in *alf* mutants. In support of this mechanism, we consistently find labeled mesenchymal cells in the distal-most regions of overgrown tissue in mosaic animals.

AER signaling in amniotes requires connexin-mediated electrical connectivity between cells to coordinate pattern and growth of the vertebrate limb [65]–[67]. An analogous mechanism may be functioning in fish. We show here that altering ionic communication in the developing fin of the zebrafish is sufficient to induce growth. Our analysis of the genetic interactions between *alf* and *sof* indicate that Kcnk5b and Cx43 may act in parallel pathways to modulate final fin size. In both mutants segment length and fin size are correlated, however the role of segment patterning in size regulation of the fin is unclear. In contrast to *alf* [36], [68] (Figure 1) and *sof* [33], the overgrowth mutants *lof* and *rpz* have wild type sized lepidotrichial segments [32]. Moreover, the *evx1* mutation, which leads to fins rays devoid of joints, does not affect final fin size in a wild type nor *lof* background [69].

### Ionic currents and positional information

Several experiments suggest that bioelectrical signaling is a shared common mechanism used across bilaterians to control organ growth and patterning [21], [70], [71] and indicate that ion flow may have an instructive role during development [72], [73], as well as regeneration [18], [74]. Here, we provide genetic evidence showing that changes in K^+^ channel activity result in allometric scaling of an organ, rather than causing uncontrolled proliferation. We favor a model for size regulation in which modulation of ionic current by K^+^ channels within the organ shifts positional information, thereby setting a different register of size during development. In fact, there is evidence for a rostro-caudal and medio-lateral gradient of voltage within the developing embryo suggesting that electric fields are a component of the positional information [75], [76]. External electrical currents have been shown to alter positional information in axial regenerates of planaria [77]. However, the underlying mechanism of signaling from electrical fields is largely unknown, and possibly depends on electrical coupling between cells [78]. This hypothesis is supported by studies in pigment pattern formation, where both K^+^ channels and connexins have been implicated in proper formation of the zebrafish stripes [49], [79]–[81]. Further studies will be needed to uncover the signaling mechanism from K^+^ channels to regulate size and pattern. However, our work, in concert with that of others, clearly shows that ion flow is not just an epiphenomenal event accompanying growth but one of the major factors specifying pattern and form during development and regeneration.

## Materials and Methods

### Fish maintenance

Zebrafish were bred and maintained as previously described [82]. *alf^dty86d^* was isolated in the 1996 Tübingen screen [30], [34] as a mutant affecting adult fin formation. The *alf^dt30mh^*(*pfau*) mutant was identified in F1 fish of a standard F3 screen (ZF Models) and isolated based on its fin and barbel phenotype.

### Measurements

Fish were anesthetized in tricaine solution for measurements; fin length and standard length was measured using handheld calipers. Fish were imaged under a stereo microscope (Zeiss, SteREO Discovery) and measurements were performed using AxioVision software (Zeiss). p-values from unpaired Student's t-test were obtained with Microsoft Excel.

### Sections and PCNA staining

Fin regenerates were fixed at 4 dpa in 4% PFA overnight and decalcified with 0.5 M EDTA for 24 h. Sample were embedded in paraffin and sectioned at 5 µm. Immunohistochemistry with anti-PCNA antibody (Sigma) was performed as described [83]. Percentage of PCNA positive nuclei over Hoechst positive nuclei was determined on three to four sections of four independent samples for each genotype.

### RT-PCR analysis

Adult zebrafish organs were dissected on ice and stored in RNALater (Invitrogen) at 4°C. Total RNA was isolated using RNeasy Mini kit (Qiagen). cDNA was synthesized from 200 ng RNA from each sample with SuperScript III and oligo(dT) primers (Invitrogen). PCR analysis was performed using Taq polymerase S (Genaxxon) with intron spanning primers (β-actin forward OSP-31, TGC GGA ATA TCA TCT GCT TG, β-actin reverse OSP-32: AGC ATC ATC TCC AGC GAA TC, *kcnk5b* forward OSP-390: CAT TCC TCT GTG CCT CAC CT; *kcnk5b* reverse OSP-324 AGG CCA TCC ACA GAC TCA TC, T_m_ = 61°C, 30 cycles).

### Mapping

Mapping was performed as described [82]. The *alf^dty86d^* mutation mapped between z11841 (5 recombinants/96 meioses) and z21067 (2/96) and fine mapped using SNPs. *alf^dt30mh^* mapped between z7803 (1/48) and z21067 (1/48). Full length *kcnk5b* was cloned into pGEM-T Easy from cDNA of fin blastema amplified with LA Taq polymerase (TaKaRa) (forward primer OSP-379: TGG GAG TGT GGA GTG TGT GT, reverse OSP-382: TTT TTG GTC CAG CTT TGG TC, T_m_ = 60°C, 45 cycles).

### X-ray irradiation and screening for revertants of *alf*

Sperm from *alf^dty86d^* homozygotes was irradiated with X-rays (1125 rads, Faxitron 43855D) and used to fertilize wild type eggs (AB strain). F1 progeny was reared to approximately three weeks of age (9433 fish) and screened for the *alf* phenotype. 11 fish showed wild type fins. 10 of these survived to adult stages. SSLP analysis revealed that 9 of these were deletions of some or all of the upper arm of chromosome 20. q-RT-PCR of candidate genes in the remaining revertant (j131x8) showed no change in transcript levels for *bpnt*, *ylpm1* and *syt14*, but little or no transcript for *kcnk5b*. PCR analysis of genomic DNA showed that this revertant has a 384 bp deletion of the 3′ end of intron 2 and the 5′ end of exon 3. This deletion results in a frameshift and early truncation of the protein.

### Modeling of Kcnk5b

The amino acid sequence of zebrafish Kcnk5b was retrieved from Ensembl (http://www.ensembl.org) and used to search the PDB database with HHpred (http://toolkit.tuebingen.mpg.de/) [84]. The first hit in the search (human KCNK4, PDB ID: 3um7 [39] identity 36%, similarity 0.646; 22^nd^ March 2012) was used to build the 3D model. The model was processed with MacPyMol (http://pymol.org).

### Electrophysiological measurements

*kcnk5b* was subcloned from pGEM-T Easy to pSGEM expression vector via *Sac*II and *Spe*I sites. After linearization with *Nhe*I, cRNA was synthesized with Ambion mMessage mMachine (Invitrogen) and cleaned up with mRNeasy Mini Kit (Qiagen). *X. laevis* oocytes were injected as described previously [85] (*kcnk5b* single alleles: 4 ng wild type or mutant *kcnk5b* cRNA; co-injections of two *kcnk5b* alleles: 2 ng cRNA each, for a total of 4 ng per oocyte). Measurements were done from a holding potential of −80 mV with 0.5 s long pulses from −100 to +60 mV with increments of 20 mV. Recorded currents (n = 5–26) were averaged and normalized to the mean value recorded for oocytes injected with the wild type channel at +60 mV.

### Generation of the *kcnk5b*(GFGAAA) non-conductive mutant

PCR mutagenesis was performed as described [86] using Pfu polymerase (Fermentas) (OSP-15 CCC TGA CGA CTG TCG CTG CAG CTG ACT ATG TGG CAG GGG C; OSP-16 CCT GCC ACA TAG TCA GCT GCA GCG ACA GTC GTC AGG GTG G, Tm = 70°C, 30 cycles) on pSGEM:kcnk5b(wt).

### Cloning of overexpression vectors

***ef1a:DsRed*** **vector**. A *ef1a:DsRed* cassette generated with KOD Hot Start DNA Polymerase (Toyobo) (primers: TAA TTT AAA TAG ATC TTC GAG CAG GGG GAT CAT CTA ATC A; CTA GAT GGC CAG ATC TGC CCG GGA CTT GAT TAG GGT GAT GGT TCA CGT AGT G, T_m_ = 59°C, 30 cycles) from plasmid Ale237 (kind gift of Alessandro Mongera) was inserted in plasmid 587jk (kind gift of Dr. Jana Krauß) using *Bgl*II restriction sites through In-Fusion Advantage (Clontech) cloning according to manufacturer's protocol.

***ef1a:DsRed; ef1a:kcnk5b*** **wild type and mutant vectors**. The *ef1a* promoter was amplified from plasmid Ale237 (primers: ATT AAT TCG AGC TCG GTA CCC CTC GAG CAG GGG GAT CAT CT; GAA CAA GCA AGC TGG GTA CCC CGG CCG TCG AGG AAT TCT TTG, T_m_ = 59°C, 30 cycles) and inserted into the pSGEM vector at the *Kpn*I restriction site using In-Fusion Advantage (Clontech) cloning. The *ef1a:kcnk5b* cassette was amplified from the resulting plasmid as above (primer: AAA CCT AGG TCG AGC AGG GGG ATC ATC T; AAA CCT AGG ATG ACC ATG ATT ACG CCA AGC TAT), digested with *Avr*II and inserted into *ef1a:DsRed* vector using the *Spe*I restriction site.

### Injections

Plasmids (5–20 ng/µl), Tol2 mRNA (25 ng/µl) and 20% (v/v) phenol red solution (Sigma- Aldrich, P0290-100ML) were injected into the zygote of 1-cell stage embryos under a dissecting microscope (Zeiss, Stemi 2000) using 275 Pa (40 psi) injecting pressure for 100 ms (World Precision Instruments, Pneumatic PicoPump PV820). Adults were analyzed with Zeiss, SteREO Discovery and Zeiss LSM 5 Live.

### Transplantations

Transplantations were performed as previously described [82]. At mid blastula stage (1000 cell stage), about 20–40 cells were transplanted from the *pfau^dt30mh/+^* donors into the recipient close to the yolk cell and chimeras were raised to adulthood.

### *In vivo* analysis of membrane potential

Fluorescent dye experiments were performed by adapting described protocols [22], [87]. Briefly, wild type and mutant juvenile fish (STL = 16–18 mm) were incubated in fluorescent dye diluted 1∶2000 in fish water (stock solutions: DiSBAC_2_(3) (Bis-(1,3-Diethylthiobarbituric Acid)Trimethine Oxonol, Life Technologies): 1 mg/ml in DMSO) for 30 min in the dark, anesthetized with tricaine solution and placed on a custom-made chamber for confocal imaging. The chamber was obtained by removing the bottom of a 55 mm plastic dish and by replacing it through a round cover slip fastened with silicone. Fish were held in place with a tissue soaked in dye and imaged upon excitation at 561 nm. Unstained animals were imaged as a negative control. p-values from unpaired Student's t-test were obtained with Microsoft Excel.

## Supporting Information

Figure S1 Phenotype of homozygous *alf* mutants. (A) wt, (B) *alf^dty86d^* homozygous, (C) *alf^dt30mh^* homozygous. Scale bar: 10 mm
(TIF)

Figure S2 *kcnk5b* gain-of-function mutations affect local growth of appendages. (A) Transplantation of *kcnk5b^dt30mh^* ^/+^ cells into wt *albino* hosts. If the mutation acts on a systemic level, mutant clones should promote overgrowth of all appendages. If the mutation has a local effect, overgrowth will be observed in patches. Chimeras resulting from the transplantation experiments show overgrowth of (B) single fins, (C) fin parts or (D) individual barbels.
(TIF)

Figure S3 The control plasmid *ef1a:DsRed* drives DsRed expression in a wide range of cell types and tissues within the fin. (A) lateral line, (B) vasculature, (C) osteoblasts, (D) fibroblasts, (E) pigment cells (arrows), showing the typical stellated shape, and (F) epidermis. Scale bar: 200 µm
(TIF)

Figure S4 Polarization of fins during growth. Voltage sensitive dyes were used to assess changes in overall polarization of growing caudal fins of wild type and *alf* juvenile fish. (A) DiSBAC_2_(3) staining in wild type fins exhibited hyperpolarization localized to discrete regions of the fin with variable detection of distal regions of altered depolarization. (B) *alf* fins in contrast show high levels of depolarization across the fin with variable patterns in different tissues. (C) Quantification of average DiSBAC_2_(3) fluorescence signal in wild type and mutant fins (average pixel intensity (12-bits) of the fin in maximum intensity projections). ***: p<0.001, N = 21–23. (D) Positive control of depolarization by treatment of the fins with 150 mM KCl (D′). DiBAC_4_(3), another dye sensitive to depolarization, showed similar effects, while DiSC_3_(5), a dye sensitive to hyperpolarized states, was uninformative (data not shown).
(TIF)
