# Supplementary figures and images for: Bioelectric Signaling Regulates Size in Zebrafish Fins
Source: PLoS Genet. 2014 Jan 16;10(1):e1004080. doi: 10.1371/journal.pgen.1004080 (PMC3894163; doi:10.1371/journal.pgen.1004080)

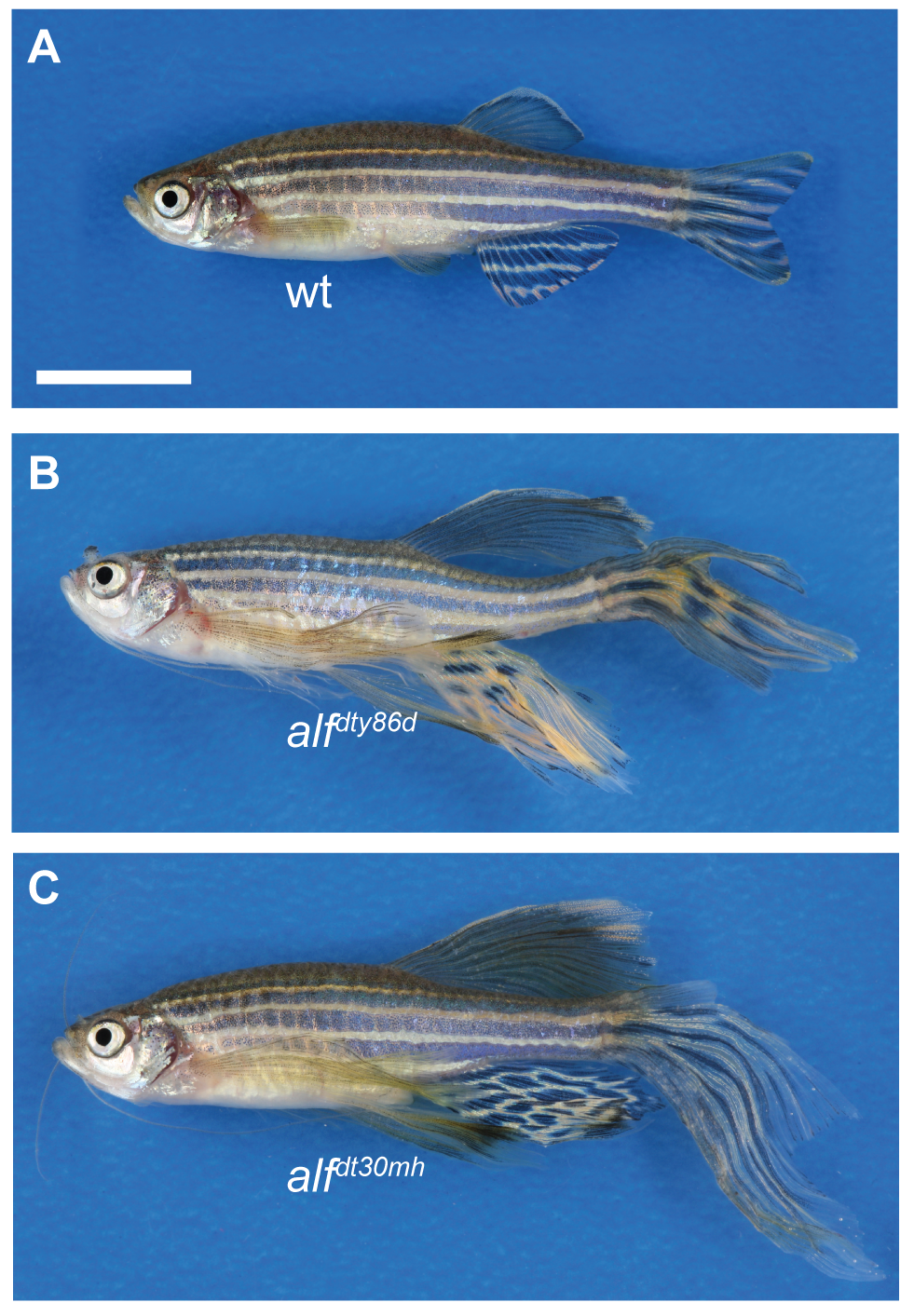

Supplement: Figure S1 — Phenotype of homozygous alf mutants. (A) wt, (B) alfdty86d homozygous, (C) alfdt30mh homozygous. Scale bar: 10 mm (TIF) [file pgen.1004080.s001.tif]

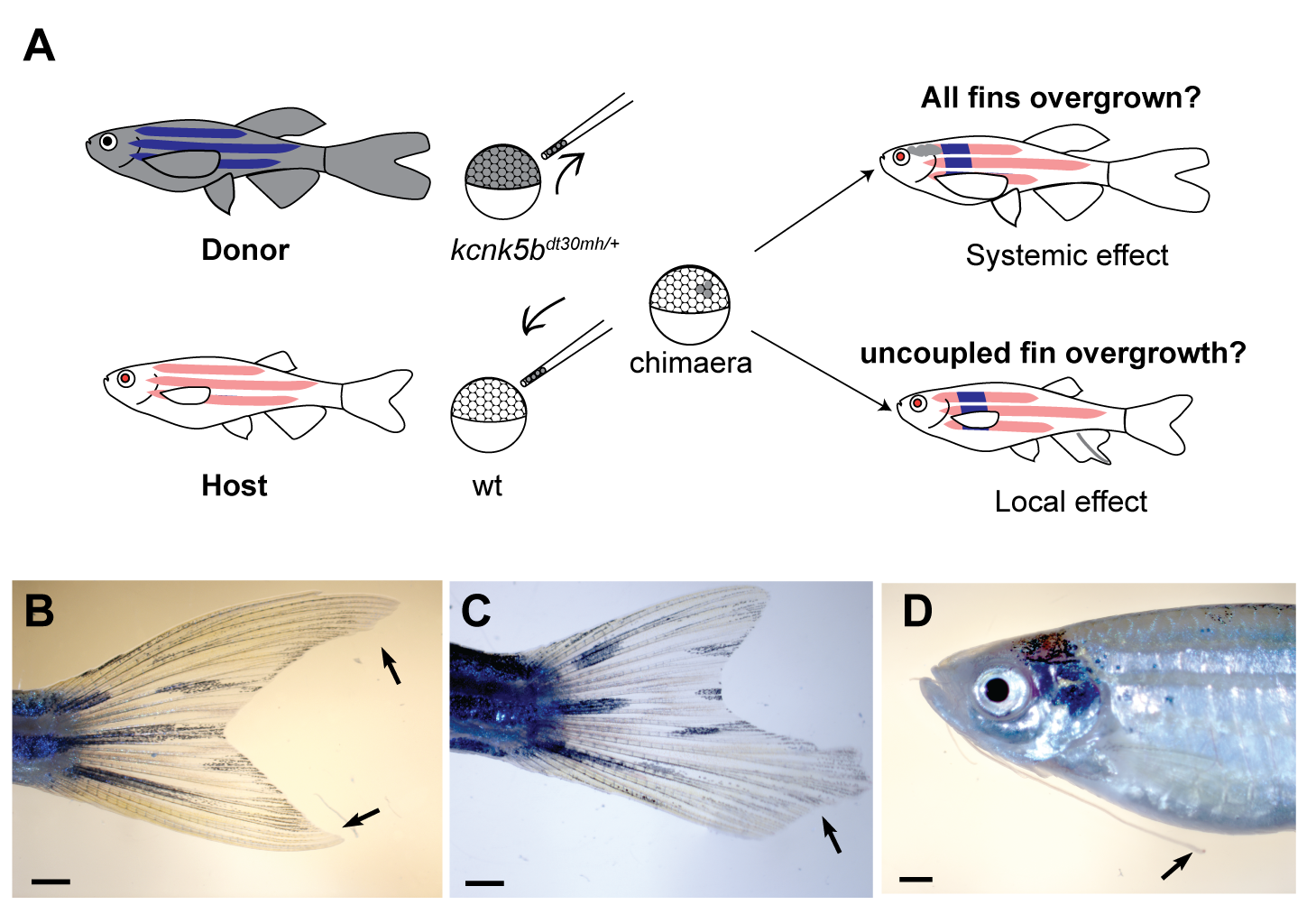

Supplement: Figure S2 — kcnk5b gain-of-function mutations affect local growth of appendages. (A) Transplantation of kcnk5bdt30mh /+ cells into wt albino hosts. If the mutation acts on a systemic level, mutant clones should promote overgrowth of all appendages. If the mutation has a local effect, overgrowth will be observed in patches. Chimeras resulting from the transplantation experiments show overgrowth of (B) single fins, (C) fin parts or (D) individual barbels. (TIF) [file pgen.1004080.s002.tif]

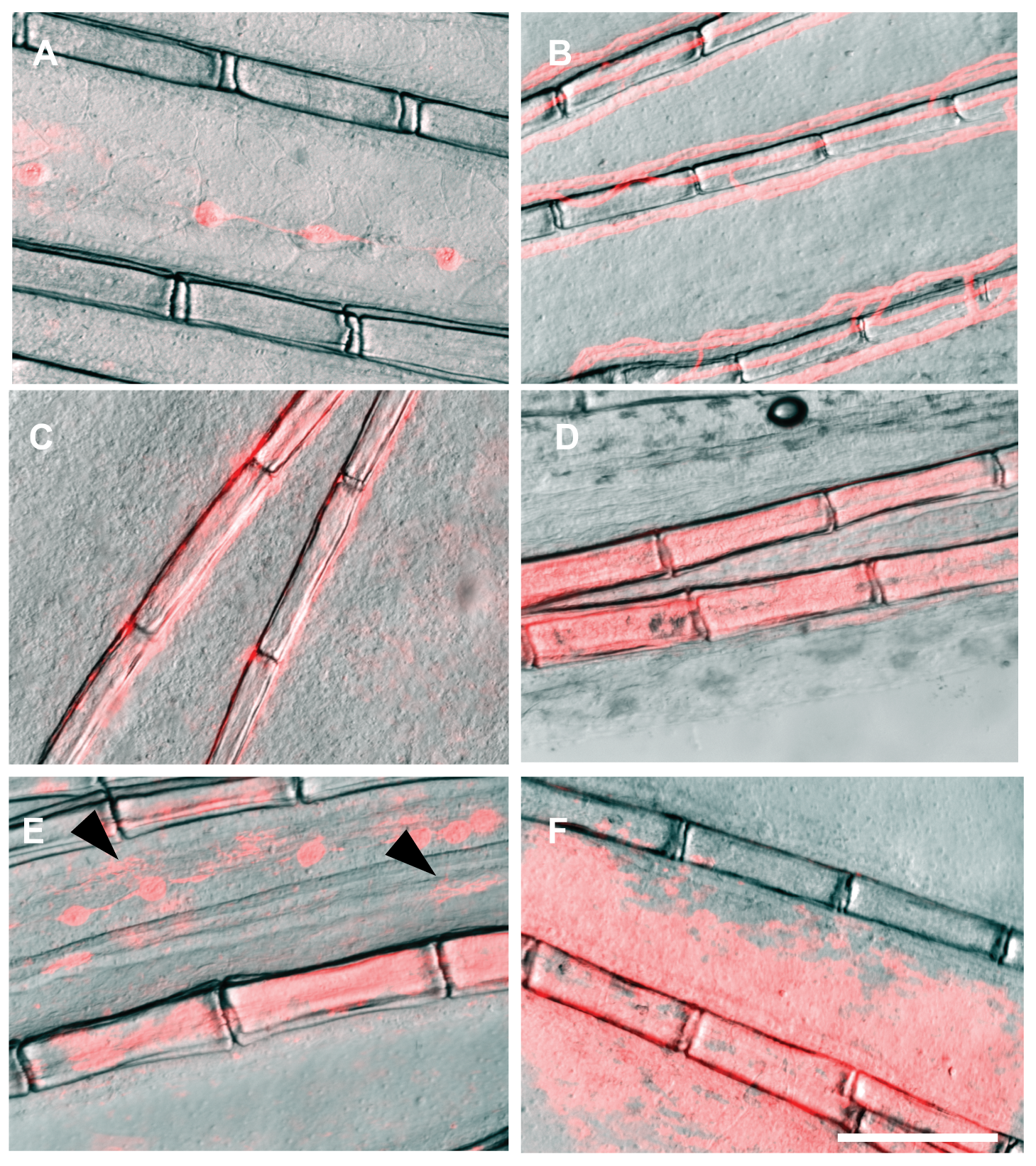

Supplement: Figure S3 — The control plasmid ef1a:DsRed drives DsRed expression in a wide range of cell types and tissues within the fin. (A) lateral line, (B) vasculature, (C) osteoblasts, (D) fibroblasts, (E) pigment cells (arrows), showing the typical stellated shape, and (F) epidermis. Scale bar: 200 µm (TIF) [file pgen.1004080.s003.tif]

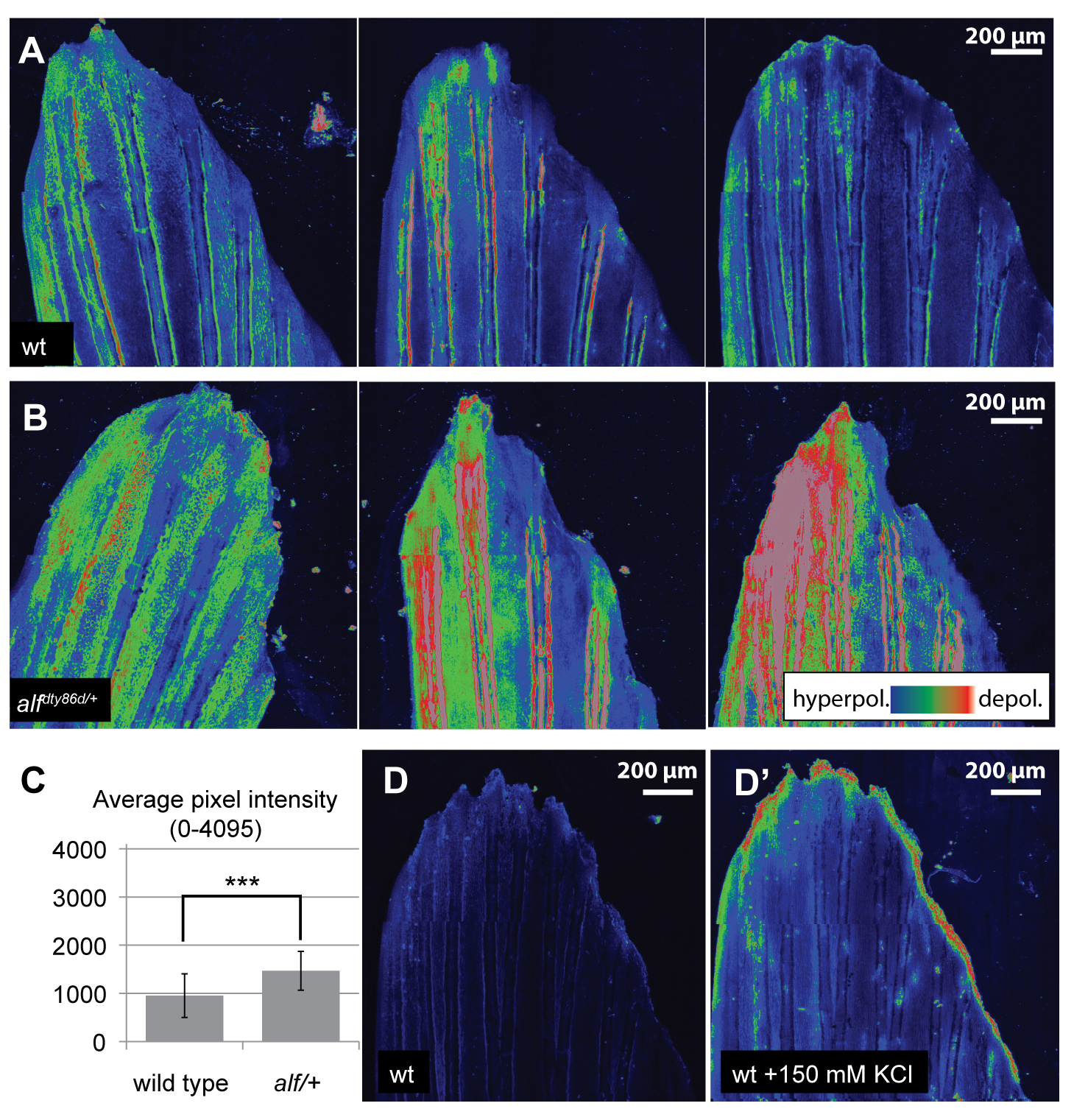

Supplement: Figure S4 — Polarization of fins during growth. Voltage sensitive dyes were used to assess changes in overall polarization of growing caudal fins of wild type and alf juvenile fish. (A) DiSBAC2(3) staining in wild type fins exhibited hyperpolarization localized to discrete regions of the fin with variable detection of distal regions of altered depolarization. (B) alf fins in contrast show high levels of depolarization across the fin with variable patterns in different tissues. (C) Quantification of average DiSBAC2(3) fluorescence signal in wild type and mutant fins (average pixel intensity (12-bits) of the fin in maximum intensity projections). ***: p<0.001, N = 21–23. (D) Positive control of depolarization by treatment of the fins with 150 mM KCl (D′). DiBAC4(3), another dye sensitive to depolarization, showed similar effects, while DiSC3(5), a dye sensitive to hyperpolarized states, was uninformative (data not shown). (TIF) [file pgen.1004080.s004.tif]
